# Bactericidal/Permeability-Increasing Protein Preeminently Mediates Clearance of *Pseudomonas aeruginosa In Vivo via* CD18-Dependent Phagocytosis

**DOI:** 10.3389/fimmu.2021.659523

**Published:** 2021-04-26

**Authors:** Jomkuan Theprungsirikul, Sladjana Skopelja-Gardner, Ashley S. Burns, Rachel M. Wierzbicki, William F. C. Rigby

**Affiliations:** ^1^ Department of Microbiology and Immunology, Geisel School of Medicine at Dartmouth, Lebanon, NH, United States; ^2^ Division of Rheumatology, Department of Medicine, Geisel School of Medicine at Dartmouth, Lebanon, NH, United States

**Keywords:** Bactericidal/permeability-increasing protein (BPI), *Pseudomonas*, phagocytosis, CD18, neutrophils, inflammation, innate immunity, opsonization

## Abstract

Chronic *Pseudomonas aeruginosa* infection mysteriously occurs in the airways of patients with cystic fibrosis (CF), bronchiectasis (BE), and chronic obstructive pulmonary disease (COPD) in the absence of neutrophil dysfunction or neutropenia and is strongly associated with autoimmunity to bactericidal permeability-increasing protein (BPI). Here, we define a critical role for BPI in *in vivo* immunity against *P. aeruginosa.* Wild type and BPI-deficient (*Bpi-/-)* mice were infected with *P. aeruginosa*, and bacterial clearance, cell infiltrates, cytokine production, and *in vivo* phagocytosis were quantified. *Bpi-/-* mice exhibited a decreased ability to clear *P. aeruginosa in vivo* in concert with increased neutrophil counts and cytokine release. *Bpi-/-* neutrophils displayed decreased phagocytosis that was corrected by exogenous BPI *in vitro*. Exogenous BPI also enhanced clearance of *P. aeruginosa* in *Bpi*-/- mice *in vivo* by increasing *P. aeruginosa* uptake by neutrophils in a CD18-dependent manner. These data indicate that BPI plays an essential role in innate immunity against *P. aeruginosa* through its opsonic activity and suggest that perturbations in BPI levels or function may contribute to chronic lung infection with *P. aeruginosa*.

## Introduction

Polymorphonuclear cells or neutrophils (PMNs) are an essential component of innate immunity against *Pseudomonas aeruginosa* infection (1, 2). Neutropenic mice are more susceptible to *P. aeruginosa*-related mortality (2), while humans deficient in neutrophil-mediated antimicrobial mechanisms (typically altered phagocytic function) have markedly increased susceptibility to *P. aeruginosa* infection (3, 4). Thus, immunity against *P. aeruginosa* is dependent on neutrophil-mediated phagocytosis. Chronic *P. aeruginosa* infection occurs in the airways of patients with cystic fibrosis (CF), non-CF bronchiectasis (BE), and chronic obstructive pulmonary disease (COPD) in the absence of neutrophil dysfunction or neutropenia (5–10). The underlying mechanism(s) of chronic *P. aeruginosa* airway infection in the context of intact neutrophil function is unknown. We previously reported a strong association between autoreactivity to bactericidal/permeability-increasing protein (BPI) and chronic *P. aeruginosa* infection in the serum of CF and BE patients (5, 6). Moreover, levels of autoantibodies to BPI inversely correlate with patient lung function in CF, BE, and COPD (5–9).

In an infected airway, neutrophils mediate killing of *P. aeruginosa* through phagocytosis, neutrophil extracellular trap (NET) formation, and neutrophil degranulation to release bactericidal proteases (11–13). BPI is an antimicrobial agent within neutrophil azurophilic granules that mediates membrane injury of gram-negative bacteria (GNB) following its high affinity binding to lipopolysaccharides (LPS) (14–16). This bactericidal activity of BPI has been recognized *in vitro* and is transduced by the N-terminal BPI domain (15, 17–22). Mouse BPI shows 53% amino acid identity and 71% similarity to human BPI; overexpression of mouse BPI in human embryonic kidney 293 cells resulted in antibacterial activity against *Escherichia coli*, comparable with that obtained with human BPI (23). The ability of BPI to mediate *E. coli* opsonization has been reported *in vitro* and attributed to the ability of the BPI C-terminal domain to engage myeloid cells through an undetermined pathway (17). The function of BPI in innate immunity against GNB *in vivo* has not been studied.

We demonstrated that BPI is required for clearance of *P. aeruginosa in vivo*, in both lung and peritoneal infection models. The absence of BPI resulted in increased inflammation, characterized by enhanced neutrophil recruitment and inflammatory cytokine production in both the lung and the peritoneum. In the absence of BPI, altered *P. aeruginosa* clearance was due to impaired phagocytosis of *P. aeruginosa* by BPI-deficient neutrophils and not due to an impairment in degranulation or production of reactive oxygen species (ROS). Exogenous human BPI decreased bacterial load and reduced inflammation *in vivo* by facilitating *P. aeruginosa* uptake into *Bpi-/-* neutrophils, in a CD18-dependent manner. Together, these findings provide the first *in vivo* evidence that BPI is essential for eliminating *P. aeruginosa* infection. Moreover, this activity of BPI is transduced through enhanced phagocytosis of *P. aeruginosa*, rather than by direct bacterial cytotoxicity.

## Methods

### Mice

Age-matched male and female (6-10 weeks old) mouse littermates on the C57BL/6 background were used for all experiments. Heterozygous *Bpi+/-* (C57BL/6NJ-Bpi^em1(IMPC)J^/Mmjax) mice were obtained from The Jackson Laboratory (MMRRC Stock: 42125-JAX, Bar Harbor, ME) and bred in our facility. This study was carried out in strict accordance with the recommendations in the Guide for the Care and Use of Laboratory Animals of the National Research Council (24). All experiments were conducted according to protocols approved by Dartmouth College’s Institutional Animal Care and Use Committee (IACUC Protocol 00002007). Tail DNA genotyping was performed on pups between 8-14 days of age (**Supplemental Figure 1**) following the specific strain protocol provided by The Jackson Lab (MMRRC Stock: 42125-JAX). No developmental phenotype was observed with homozygous *Bpi-/-* mice.

### Murine Neutrophil Isolation and Lysate Generation

For isolation of murine neutrophils, naïve mice (wt or *Bpi*-/-) were injected intraperitoneally (i.p.) with 1 ml of 4% thioglycollate solution. Cells were harvested by peritoneal lavage (3ml PBS) 12–18 h later, a time of peak neutrophil induction (25, 26). Cells were spun down (1000rpm for 5mins) and erythrocyte lysis performed using 1X BD Pharm Lyse (BD Bioscience). To generate cell lysate, neutrophil pellet was resuspended in citrate-phosphate buffer (0.2M Na_2_HPO_4_ + 0.1M citric acid, pH 3.0) and frozen/thawed three times (6). Lysate protein concentration was determined by Pierce BCA Protein Assay Kit (ThermoFisher).

### BPI Detection by Immunoblot and Immunofluorescence (IF)

Immunoblotting against mouse or human BPI (0.5 μg purified mBPI or 20 μg mouse neutrophil protein extract, generated by repeated freeze-thaw cycles in citrate-phosphate buffer) was performed after resolution by SDS-PAGE (12% acrylamide gel) and transfer to a nitrocellulose membrane. Following blocking in Tris buffered saline with 0.05% tween-20 (TBST) + 3% bovine serum albumin (BSA), the membranes were probed with affinity purified mouse BPI antibody (POCONO, amino acid 256-269, 0.1μg/ml), anti-hBPI antibody (Santa Cruz, amino acid 227-254, 1:1000) or mouse beta actin antibody (Cell Signaling, 1:1000) in TBS-T + 1% BSA overnight at 4°C, washed, and incubated with goat anti-mouse or anti-rabbit peroxidase-labeled secondary antibody (1:50000 in TBS-T + 1% BSA). Secondary antibody-only blots were performed as controls (no signal was detected). SuperSignal West Pico (ThermoFisher) was used for protein detection *via* the Syngene G-Box system and software (Synoptics).

To detect mouse BPI by IF, thioglycolate-elicited peritoneal neutrophils (250,000 cells in 0.5 ml RPMI+10% FBS) that had been collected by lavage were plated onto 13 mm coverslips for 1 h. Samples were fixed with 4% paraformaldehyde (PFA), washed, permeabilized using 0.5% Triton X-100, and blocked using 5% donkey serum (Sigma-Aldrich). Mouse BPI was detected using rabbit anti-mouse BPI antibody (1:200, ABclonal), followed by donkey anti-rabbit Alexa Fluor 647 or donkey anti-rabbit Alexa Fluor 488 (1:500, Abcam). Samples were mounted using ProLong Gold Antifade Mount with DAPI (ThermoFisher Scientific) and visualized with the laser point scanning confocal microscope (ZEISS LSM 800, Zeiss), 63X. To detect human BPI by IF from *in vivo* administration of BPI, peritoneal cells were plated onto 13 mm coverslips, fixed, permeabilized, and blocked as mentioned above. Human BPI was detected using anti-human BPI antibody against aa227-254 (1:200, Santa Cruz), followed by donkey anti-mouse Alexa Fluor 488 (1:500, Jackson ImmunoResearch).

### Bacterial Culture and Phagocytosis Assays

*P. aeruginosa* strains PAO1 and PA14 (wt and ΔpopB) were obtained from Dr. Brent Berwin. Bacteria were cultured overnight in lysogeny broth (LB) at 37°C and sub-cultured for 3hrs in LB (1:30) at 37°C. Phagocytosis assay of live *P. aeruginosa* bacteria was performed as previously described (27, 28). Neutrophils (250,000 in 250ul RPMI, with or without 30mins 37°C pre-incubation with human or mouse BPI) were incubated with *P. aeruginosa* (PAO1 or PA14) for 45mins at 37°C at 10 or 25 multiplicities of infection (MOI) and then treated with 250ul of gentamicin (200μg/ml) for 20mins at 37°C to kill extracellular bacteria before being pelleted and washed twice in 500ul RPMI. Neutrophils were resuspended in lysis buffer (0.1% Triton X-100 in PBS) and lysed contents (10ul) plated on 1.5% LB agar plate and incubated overnight at 37°C. Bacterial colonies (colony-forming unit, CFU) were counted on the following day, representing the number of live bacteria phagocytosed by the neutrophils. To confirm phagocytosis, neutrophils were treated with GFP-expressing *P. aeruginosa* (PAO1) as above. After incubation with gentamicin and washing step, 200ul of lysis buffer was added to the tubes, mixed, and transferred to a 96-well Flat Clear Bottom Black Polystyrene Microplates (Fisher Scientific). Fluorescence was measured with BioTek Synergy plate reader (485/528 nm).

### BPI-LPS Neutralization Assay

Thioglycollate-recruited murine neutrophils obtained by peritoneal lavage (wt or *Bpi-/-*, 200,000 cells/well in 50ul DMEM) were treated with purified human BPI (Athens Research and Technology; 0, 100, 300, or 600nM) 30mins at 37°C prior to addition of *E. coli* LPS (0.1 ng/ml, final volume 200ul). Cells were incubated for 4hrs, spun down, and supernatants collected (−80°C). TNFa concentration was analyzed by mouse TNFa ELISA MAX Deluxe Set (BioLegend).

### Intraperitoneal and Oropharyngeal *P. aeruginosa In Vivo* Challenge and Cytokine Analysis

For *in vivo* intraperitoneal (i.p.) and oropharyngeal exposure sublethal infection, mice (wt or *Bpi*-/-) were anesthetized with isoflurane (to effect) and either injected with 3x10^6^ CFU of sub-cultured PA14 or PBS i.p. (300ul) or by oropharyngeal administration (50ul). For those receiving BPI treatment, 10μg of human BPI in PBS (Athens Research) was injected i.p. 15mins following bacterial infection. Mice were euthanized 3hrs post-infection (hpi). Peritoneal and bronchoalveolar lavage (BAL) fluids were collected using 1.5ml and 0.8ml PBS, respectively and 10ul plated (1:10 dilution peritoneal lavage; no dilution BAL) on 1.5% LB agar plate. Plates were incubated overnight at 37°C and recovered CFUs determined by colony counts that were back-calculated to the original lavage volume. Excess peritoneal and BAL samples were spun down (3000rpm 5 mins) and cell-free lavages stored at −80°C for cytokine analysis. Lysates from cell pellets were made in citrate-phosphate buffer as mentioned above and stored for immunoblotting. Blood samples were collected from the inferior vena cava and serum extracted. TNFα, IL-1β, and IL-6 concentrations in the lavage and serum samples were determined with mouse ELISAs: TNFa MAX Deluxe Set (BioLegend), IL-1β Duoset, and IL-6 Duoset (R&D Systems).

### Quantification of Neutrophils and MPO From *In Vivo* Challenge

Following *in vivo* challenge, cells from collected lavages were brought up in PBS to count and stained for flow cytometry analysis. Cells were washed in 1ml of PBS + 1% BSA (10^6^ cells/100ul, treated with 500ul mouse Fc Block (1:200, 10 min, 4°C), and stained (30 min, 4°C) with mouse specific antibodies (IgG) purchased from Biolegend or Invitrogen (anti-CD45-FITC (1:400), anti-Ly6C-APC (1:200), anti-Ly6G-PE (1:200), anti-CD11b-PerCP-Cy5.5 (1:200)). Cells were fixed in 1% PFA and analyze by flow cytometry (Gallios, with Kaluza Analysis or FlowJo software). Compensation was done using UltraComp eBeads (Invitrogen). Number of neutrophils (CD45+CD11b+ Ly6C^int^Ly6G^hi^) in lavage samples was determined based on total cell count in the samples, normalized to volume collected. Data from BAL was presented as %*Bpi-/-*. Mouse Myeloperoxidase DuoSet ELISA (R&D Systems) was used to measure MPO concentration in lavage samples (1:10).

Murine hind limb long bone dissection and bone marrow isolation was performed as described previously (29). Briefly, mice were euthanized using CO_2_ according to IACUC protocol and guidelines. Following femoral extraction, bone marrow cells were flushed out with cold PBS and a 22-gauge needle. Cells from the perfused bone marrow were passed through a 70-μm filter cell strainer. Erythrocytes were lysed using 1X BD Pharm Lyse (BD Bioscience). Staining and flow cytometry were performed as mentioned above.

### *In Vivo* Phagocytosis Blocking *via* CD18 Receptor

Blocking of phagocytosis was achieved by i.p. injection of anti-CD18 M18/2 antibody or a rat IgG2b isotype control (50μg, Bio X Cell) 15 mins after mice were infected with 3x10^6^ CFU of *P. aeruginosa* PA14 in 200ul PBS. In *Bpi-/-* mice, BPI (10μg) was administered i.p. 15 mins following the antibody injection. After 1.5hpi, i.p. lavage was performed (due to unequal immune cell-recruitment at 3hpi, shorter experiment was performed here). Cell-free lavage was plated onto LB agar plates for *P. aeruginosa* colony count. Peritoneal immune cells were counted to ensure comparable cell recruitment into the peritoneal cavity.

### Lung Histology

Mice (wt and *Bpi*-/-) infected *via* oropharyngeal route (non-invasive) with *P. aeruginosa* (PA14, 3x10^6^ in 50ul PBS, 3hpi) were sacrificed and lungs harvested and fixed in 10% formalin (Fisher Scientific) for at least 24hrs. Paraffin sections were generated and stained with Hematoxylin and eosin (H&E) by the Pathology Research Resource at Dartmouth–Hitchcock Medical Center. The slides were imaged using optical microscope (Olympus BX50), at 40x magnification.

### Quantification of Reactive Oxygen Species (ROS)

Thioglycollate-induced neutrophils from wt and *Bpi*-/- mice were stained with DCFDA dye (Cellular ROS Assay Kit, Abcam) for 30mins at 37°C in the dark, washed, and resuspended in 1X supplemented buffer (supplied with the kit) with 10% FBS. Stained neutrophils (100,000 cells/well) were left untreated or were treated with ROS inducers: tert-butyl hydrogen peroxide (TBHP, 100uM) or glucose oxidase (GO, 2U/ml) for 4hrs, 37°C. Fluorescence was measured at 485/535 nm (BioTek, Synergy HT) nm in end point mode, with sensitivity 50 and bottom read.

### Quantification of Extracellular DNA Release

Thioglycollate-induced neutrophils from wt and *Bpi*-/- mice (200,000 cells/100 μl) were treated with PA14 (MOI 10) or PAO1 (MOI 10) in DMEM without serum (unopsonized) for 3hrs, 37°C. Amount of extracellular DNA (ng) was quantified by Sytox Green (5 μM, Invitrogen) and fluorescence measurement (485/530 nm, BioTek, Synergy HT).

### Lung Tissue Homogenization

Mice were infected oropharyngeally with *P. aeruginosa* PA14wt (3x10^6^ CFU in 50ul PBS) or just PBS for 3hrs. Euthanized mouse lungs were perfused with 5 mL of ice-cold PBS injected through the right ventricle of the heart. Perfused lungs were removed and placed in RPMI on ice until processing. Lungs were minced and placed in digestion buffer of 1 mg/mL collagenase type I + 60 U DNase type I (Sigma-Aldrich) in DPBS Ca2+ Mg2+ (HyClone). Lungs were digested for 30 min at 37°C while shaking, and passed through a 70-μm filter cell strainer. Digested contents of the lungs were plated on LB agar plates to determine recovery CFU. Cells were washed (in RPMI, 1500rpm, 4°C) and residual red blood cells were lysed using 1X BD Pharm Lyse lysis buffer (BD Biosciences), for 2 min, and counted. Cells were washed and subsequently blocked using mouse Fc Block (1:200, 10 min, 4°C). Staining for flow cytometry was performed as mentioned above.

### Murine BPI (mBPI) Production and Affinity Purification of mBPI Peptide-Specific Antisera

Murine BPI plasmid was obtained from Origene (Cat. MR219864). Plasmid was transfected into TOP10 comp cells and DNA was purified using Qiagen Maxi Prep kit (Qiagen) according to manufacturer’s instruction. DNA was transfected into Expi293 cells using Expi293 Expression System Kit (Thermo Fisher Cat. A14635) according to manufacturer’s instruction. Mouse BPI was purified using Anti-FLAG Affinity Gel (Bimake, Cat. B23101) according to manufacturer’s instruction. Protein concentration was measured by the Pierce BCA Protein Assay Kit (ThermoFisher Scientific). Murine BPI was detected by immunoblotting using affinity purified rabbit anti-BPI raised against the murine-specific amino acid (256-269) antibody (Pocono Rabbit Farm & Laboratory (PRF&L), Canadensis, PA).

### *P. aeruginosa* Killing Assay by BPI

BPI (5μg/ml or 90nM) was incubated with 10^8^ CFU of PAO1 (in 100ul PBS). After 30mins at 37°C incubation, the samples were diluted 1:10000 in ice-cold LB before plated (10μl) onto LB agar plates. Plates were incubated overnight at 37°C before CFUs were counted. CFU data was normalized to no-BPI treatment (100% survival).

### Statistical Analyses

Data were analyzed using GraphPad Prism 6 software (GraphPad Software, Inc., California, USA). Student’s paired and unpaired t-tests with Welch’s correction were applied to determine the significant difference between two data sets. One-way ANOVA with multiple comparisons were applied for more than two data sets. P <0.05 was considered significant.

### Study Approval

All experiments were conducted according to protocols approved by Dartmouth College’s Institutional Animal Care and Use Committee (IACUC Protocol 00002007).

## Results

### BPI Is Required for Efficient *P. aeruginosa* Clearance and Control of Lung Inflammation

**Figure 1:**
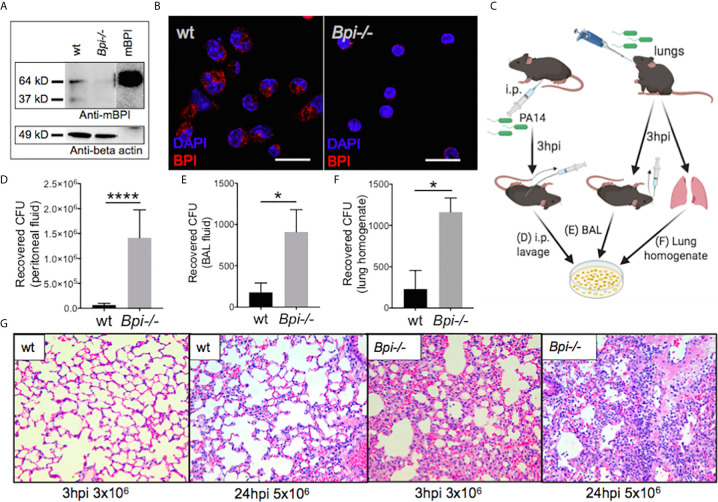
BPI is required for efficient *P. aeruginosa* clearance and control of lung inflammation *in vivo*. **(A)** Immunoblots of anti-mouse BPI (mBPI) (amino acids 256-269) and anti-beta actin antibody reactivity to neutrophil lysates (20 μg) from wild type (wt) and BPI-deficient (*Bpi*-/-) mice. Recombinant mBPI (0.5 μg) used as control. **(B)** Immunofluorescence staining of BPI (red) and DAPI (blue) in the peritoneal wt and *Bpi-/-* mouse PMNs (mPMNs). Images obtained using a 63X oil immersion objective. Scale bar: 20μm. **(C)** Mice were infected with 3x10^6^ CFU PA14 *via* intraperitoneal or oropharyngeal route before lavages or lung homogenate were obtained 3hpi to plate for colony count. **(D)** Colony forming units (CFU) recovered from peritoneal fluid of mice (n=5) infected with *P. aeruginosa* PA14 (3x10^6^ CFU) 3 hrs post-infection (hpi). **(E)** CFU recovered from bronchoalveolar lavage (BAL) of mice (n=4) infected with PA14 (3x10^6^ CFU) 3 hpi. **(F)** CFU recovered from whole lung homogenates of mice (n=3) infected with PA14 (3x10^6^ CFU) 3hpi. **(G)** Lung histology of wt and *Bpi-/-* mice infected *via* oropharyngeal route with PA14 (3x10^6^, 3hpi or 5x10^6^, 24hpi). Representative H&E staining shown at 40x magnification. Images shown are representative for three samples for each genotype. Statistical significance was determined by Unpaired t-test with Welch’s correction, ****p < 0.0001, *p < 0.05, ns, not significant; Error bars represent mean ± SEM.

We investigated the role of BPI in immunity against *P. aeruginosa*, using BPI-deficient (*Bpi*-/-) mice (**Supplemental Figure 1**). BPI deficiency was confirmed in the *Bpi*-/- neutrophil lysates, which showed no reactivity when immunoblotted with a mouse-specific BPI (mBPI) antibody, compared to neutrophils from wild type (wt) mice, which demonstrated a ~64kD band, corresponding to that seen with recombinant mBPI (**Figure 1A** and **Supplemental Figures 2A, B**). Immunofluorescence staining of thioglycollate-recruited peritoneal neutrophils confirmed cytoplasmic residence of BPI in wt and absence of BPI in *Bpi-/-* neutrophils (**Figure 1B**).

The role of BPI in the clearance of *P. aeruginosa in vivo* has not been reported. We therefore asked if BPI is required for the clearance of *P. aeruginosa* (PA14) following acute (3hr) infection. Mice, wt and *Bpi*-/-, were infected with *P. aeruginosa via* intraperitoneal or oropharyngeal routes. Colony forming units (CFU) were assayed in the lavage (peritoneal or bronchoalveolar, respectively) or lung homogenate (**Figure 1C**). Higher numbers of colony forming units (CFUs) were recovered from both the peritoneal fluid and the bronchoalveolar lavage (BAL) samples of *Bpi*-/- mice, compared to wt littermates (**Figures 1D, E**). *Bpi*-/- mice also demonstrated higher CFUs in the whole lung homogenates following acute lung infection with *P. aeruginosa* (**Figure 1F**). While no substantive differences in lung pathology were observed between the two genotypes at baseline (**Supplemental Figure 3**), the inflammatory response to *P. aeruginosa* infection in *Bpi-/-* mice was associated with increased cellularity and inflammation, compared to wt mice through H&E staining (**Figure 1G**). These findings reveal that BPI is required for *in vivo* clearance of *P. aeruginosa* and suggest that lack of *P. aeruginosa* clearance in the absence of BPI leads to excessive lung tissue inflammation.

### BPI Deficiency Enhances Neutrophil Recruitment and Cytokine Production

**Figure 2:**
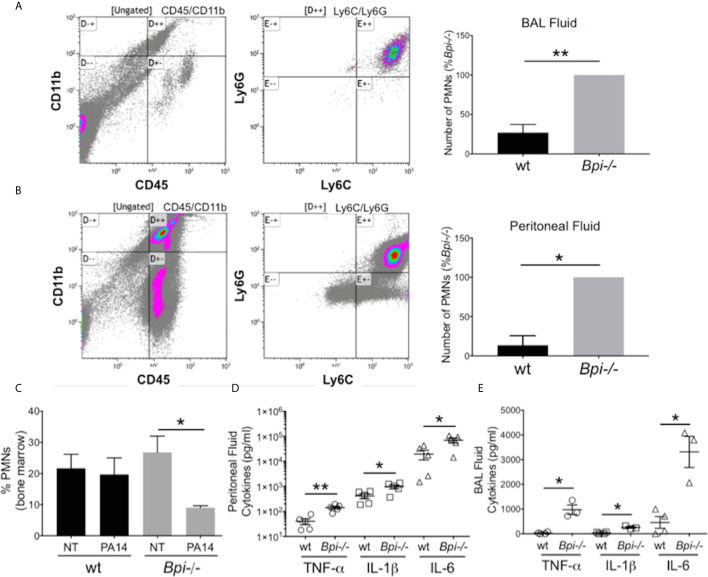
Increased neutrophil recruitment and inflammatory cytokines were observed at infected sites in BPI-deficient mice. **(A, B)** Neutrophil numbers in the **(A)** bronchoalveolar lavage and **(B)** peritoneal lavage of mice infected for 3h with PA14 3x10^6^ CFU) were determined by flow cytometry (CD45^+^CD11b^+^Ly6G^+^Ly6C^+^). Bar graphs represent number of neutrophils (PMNs) recovered from wt and *Bpi-/-* mice (n=4). Cell count was calculated from % neutrophils from flow cytometry and total number of peritoneal or BAL cells. Representative flow panels from *Bpi-/-* mice showed the majority of BAL (A, 97.3%) and peritoneal (B, 90%) immune cells are neutrophils. **(C)** Percent bone marrow neutrophils in untreated wt and *Bpi*-/- mice (n=4) as well as following oropharyngeal infection (PA14, 3x10^6^ CFU, 3hpi). **(D, E)** Inflammatory cytokines TNF, IL-1b, and IL-6 (pg/ml) were measured in **(D)** peritoneal fluid (n=5) and **(E)** BAL (n=3,4) of mice infected with PA14 (3x10^6^ CFU, 3hpi). All data were analyzed by unpaired t-test with Welch’s correction, except for data in **(C)** which were analyzed by paired Student t-test, **p < 0.01, *p < 0.05; Error bars represent mean ± SEM.

To investigate whether reduced clearance of *P. aeruginosa* in *Bpi-/-* mice was due to an impaired inflammatory response, we evaluated neutrophil recruitment to the infection site. On the contrary, significantly higher neutrophil numbers were detected in both the BAL and the peritoneum of *Bpi-/-* mice compared to wt controls following infection (**Figures 2A, B**). The enhanced neutrophil response in BPI-deficient mice was accompanied by a decrease in the percentage of neutrophils in the bone marrow of *Bpi-/-* mice (**Figure 2C**), suggesting greater infection-triggered neutrophil recruitment in the absence of BPI.

The increased neutrophil recruitment in *Bpi*-/- mice was accompanied by significantly higher concentrations of inflammatory cytokines (TNFa, IL-1b, and IL-6) in both the peritoneal and the BAL fluid samples, compared to the wt mice (up to 3.6 fold increase in peritoneal lavage and 37.4 fold increase in the BAL) (**Figures 2D, E**). No differences in the peritoneal and the BAL fluid cytokine levels were observed between wt and *Bpi*-/- mice challenged with PBS, which were overall significantly lower in the absence of PA14 infection (**Supplemental Figures 4A, B** and **Figures 2D, E**). Peritoneal infection led to significantly higher levels of serum TNFa, IL-1b, and IL-6 in *Bpi*-/-, compared to wt mice (**Supplemental Figure 5A**). With lung infection, a difference in serum cytokines was only seen with IL-1b (**Supplemental Figure 5B**). Therefore, these data demonstrate that absence of anti-microbial and anti-inflammatory functions mediated by BPI resulted in increased airway, peritoneal and systemic inflammation. Hence, not only is BPI essential for the clearance of *P. aeruginosa in vivo*, intact BPI is required for the control of the inflammatory cellular and cytokine responses during acute *P. aeruginosa* infection.

### Compromised *P. aeruginosa* Clearance *In Vivo* in the Absence of BPI Is Due to Impaired Neutrophil Phagocytosis

**Figure 3:**
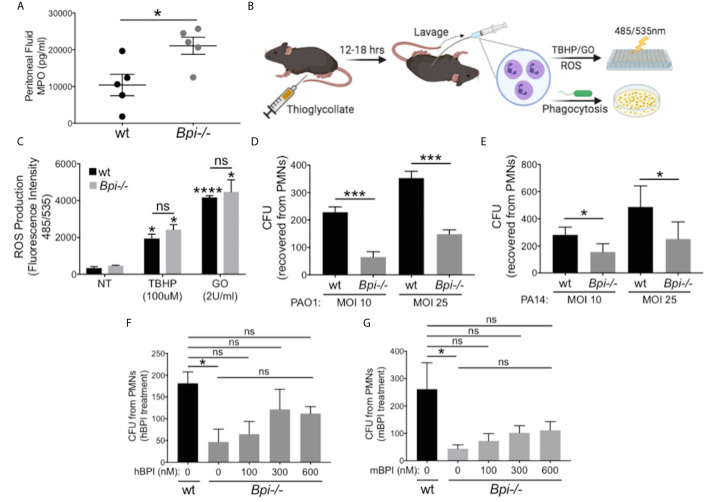
BPI is required for *P. aeruginosa* phagocytosis but not neutrophils degranulation or ROS production. **(A)** Myeloperoxidase (MPO) concentration, measured by ELISA, in peritoneal fluid of wild type (wt) and *Bpi-/-* mice (n=5) infected with PA14 (3hpi). Data were analyzed by unpaired t-test with Welch’s correction. **(B)** Mice were injected intraperitoneally with 1ml 4% thioglycollate, and neutrophils were harvested 12-18 hrs later for reactive oxygen species (ROS) or phagocytosis experiments. **(C)** ROS production of wt and *Bpi-/-* PMNs (n=3) after stimulation with tert-Butyl hydroperoxide (TBHP, 100μM) or glucose oxidase (GO, 2U/ml) for 4hrs. Statistical significance was determined relative to untreated (NT) cells (*p < 0.05) or between genotypes (ns, not significant). **(D, E)** Intracellular colony-forming unit (CFU) counts obtained from lysed wt and *Bpi-/-* mouse PMNs (mPMNs) challenged with *P. aeruginosa* **(D)** PAO1 (n=4) and **(E)** PA14 (n=3) at multiplicity of infection (MOI) of 10 and 25; Statistical significance was determined by paired Student t-test; **(F, G)** Intracellular CFU counts from wt and *Bpi-/-* mPMNs treated with *P. aeruginosa* PAO1 (MOI 25, n=3) with increasing concentrations of **(F)** native human BPI (hBPI, 0-600 nM) or **(G)** recombinant mouse BPI (mBPI, 0-600 nM). Statistical significance was determined by one-way ANOVA with multiple comparisons for **(C)**, and unpaired t-test with Welch’s correction for others. ****p < 0.0001, ***p < 0.001, *p < 0.05, ns, not significant; Error bars represent mean ± SEM.

To identify the mechanism(s) responsible for defective bacterial clearance *in vivo* in the absence of BPI, we investigated different functions of wt *vs. Bpi-/-* neutrophils, since the majority of cells recruited to infection site 3hr after infection are neutrophils (1, 2, **Figures 2A, B**, e.g. 90% in the peritoneum and 97.3% in the BAL). Degranulation was evaluated by quantifying release of myeloperoxidase (MPO), a bactericidal protein residing in neutrophil azurophilic granules alongside BPI (30). MPO levels in the peritoneal lavage fluid were higher in *Bpi-/-* compared to wt mice (**Figure 3A**), suggesting that degranulation was not compromised in *Bpi-/-* neutrophils. Thioglycollate-elicited peritoneal neutrophils were investigated *in vitro* for differences in reactive oxygen species (ROS) production or phagocytosis ability (**Figure 3B**). In agreement with their intact ability to undergo degranulation, *Bpi*-/- neutrophils produced equal levels of neutrophil extracellular traps (NETs) and ROS as the wt neutrophils upon stimulation with *P. aeruginosa* or known ROS stimuli (TBHP−tert-Butyl hydroperoxide or GO−glucose oxidase) (**Figure 3C** and **Supplemental Figure 6**). Together, these data demonstrate that compromised *P. aeruginosa* clearance in BPI-deficient mice *in vivo* is not due to impaired degranulation or ROS production by *Bpi*-/- neutrophils.

While cytocidal activity of BPI toward GNB has been reported *in vitro* (14–16, 31, 32), its role in neutrophil-mediated phagocytosis of GNB is less well understood (17, 19, 20). To investigate this, thioglycollate-elicited *Bpi*-/- and wt neutrophils were assayed for the number of ingested CFUs of *P. aeruginosa* (PAO1 or PA14) *in vitro* following gentamicin treatment to kill extracellular bacteria. Cell lysates showed significantly fewer *P. aeruginosa* CFUs were recovered from BPI-deficient neutrophils (**Figures 3D, E**). This effect was independent of the *P. aeruginosa* strain or multiplicity of infection (MOI). To confirm that CFU counts accurately represent relative phagocytic ability of the neutrophils, levels of GFP-expressing *P. aeruginosa* in neutrophil lysates were quantified by fluorescence, following gentamicin protection assay. Lower levels of GFP fluorescence intensity was detected in *Bpi-/-* neutrophil extracts relative to wt controls (**Supplemental Figure 7**). Addition of exogenous BPI (mouse or human) to *Bpi*-/- neutrophils prior to *in vitro* culture with *P. aeruginosa* increased intracellular CFU recovery in a concentration-dependent manner (**Figures 3F, G**). Moreover, the addition of exogenous BPI neutralized LPS-induced TNFa release by *Bpi-/-* neutrophils (**Supplemental Figure 8**). Together, these data suggest that BPI is required for efficient phagocytosis of *P. aeruginosa* by neutrophils *in vitro* and that it mediates anti-inflammatory properties by reducing LPS-induced TNFa release.

### Addition of BPI Enhances *P. aeruginosa* Clearance and Lowers Inflammation in *Bpi-/-* Mice *In Vivo*

**Figure 4:**
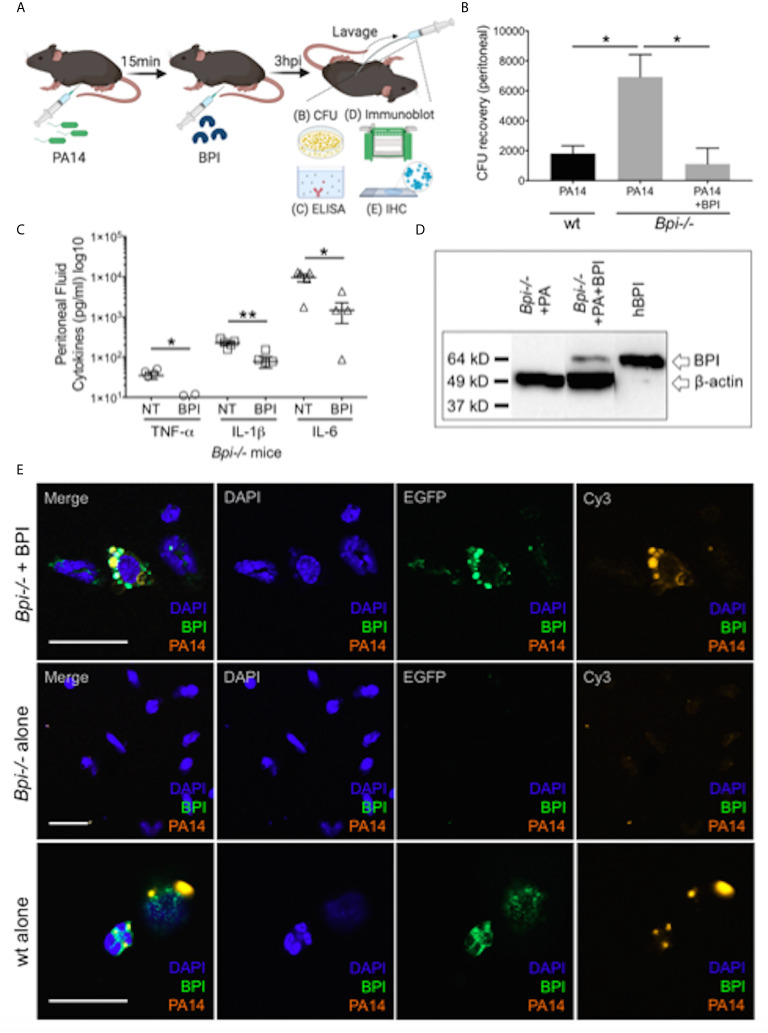
Exogenous BPI mediates *P. aeruginosa* uptake in *Bpi-/-* mice *in vivo*. **(A)** Mice were administered 10μg BPI 15mins following PA14 infection (3x10^6^ CFU) *via* intraperitoneal route. Peritoneal lavage was performed 3hpi for PA14 colony count, ELISA, immunoblot, and IHC. Administration of neutrophil-purified human BPI (hBPI, 10μg) into the peritoneal cavity of mice infected with PA14 (3x10^6^ CFUs) lowers **(B)** bacterial colony forming units (CFU) recovered from peritoneal fluid of *Bpi-/-* mice (n=4), and **(C)** concentration of pro-inflammatory cytokines found in the peritoneal fluid (n=5). Data were analyzed by unpaired t-test with Welch’s correction; **p < 0.01, *p < 0.05; Error bars represent mean ± SEM. **(D)** Immunoblot of peritoneal cell lysates (10μg protein, *Bpi-/-*) shows uptake of hBPI with anti-hBPI IgG (amino acids 227-254) following PA14 infection with or without BPI treatment *in vivo.* Anti-beta actin antibody and recombinant mBPI (0.05μg) were used as controls. **(E)** Immunofluorescence images of *Bpi-/-* peritoneal immune cells infected with td-Tomato-expressing PA14 (3x10^6^ CFU, 3hpi) with and without BPI treatment, stained with DAPI (blue) and anti-hBPI antibody (green). Fluorescent images were obtained using a 63X oil immersion objective. Scale bar: 20 μm. Images shown are representative for three samples for each treatment.

To investigate if treatment with BPI can rescue the defect in bacterial clearance seen in *Bpi-/-* mice *in vivo* (**Figures 1D–F**), we administered human BPI (hBPI, 0.2-10 μg) 15 min following intraperitoneal *P. aeruginosa* infection of *Bpi*-/- mice (**Figure 4A** and **Supplemental Figure 9**). Addition of hBPI significantly reduced *P. aeruginosa* CFU counts in the peritoneal lavage of *Bpi-/-* mice, compared to untreated controls (**Figure 4B**). CFU counts from *Bpi-/-* mice treated with hBPI were comparable to those of wt mice (**Figure 4B**). Moreover, treatment of *Bpi*-/- mice with hBPI resulted in a significant reduction in TNFa, IL-1b, and IL-6 levels in the cell-free peritoneal fluid, compared to infected *Bpi*-/- mice without hBPI treatment (**Figure 4C**). Full length human BPI (~64kD) was detected in the lysates of peritoneal immune cells (3h post infection) by immunoblotting, but not in the cells of untreated *Bpi*-/- mice (**Figure 4D** and **Supplemental Figure 2C**). Immunofluorescence staining confirmed that hBPI was internalized by the infiltrating immune cells, localizing in the cytoplasm (**Figure 4E**). Exogenous hBPI enabled uptake of *P. aeruginosa* by neutrophils [>89% of CD45+ cells in the peritoneum (**Figure 2B**)] as demonstrated by co-localization of hBPI with td-Tomato expressing *P. aeruginosa* (**Figure 4E**, top panels). No td-Tomato expressing *P. aeruginosa* was detected in the peritoneal cells of *Bpi-/-* mice without hBPI supplementation (**Figure 4E**, middle panels). Td-Tomato *P. aeruginosa* were observed in the peritoneal cells of wt mice, suggesting that the endogenous BPI also mediates *P. aeruginosa* cellular uptake *in vivo* (**Figure 4E**, bottom panels). These findings reveal that administration of exogenous BPI can rescue the ability to clear *P. aeruginosa in vivo* and suggest that a prominent function of BPI *in vivo* is to mediate phagocytosis of *P. aeruginosa*.

### BPI Mediates Clearance of *P. aeruginosa In Vivo* by Phagocytosis, Dependent on CD18

**Figure 5:**
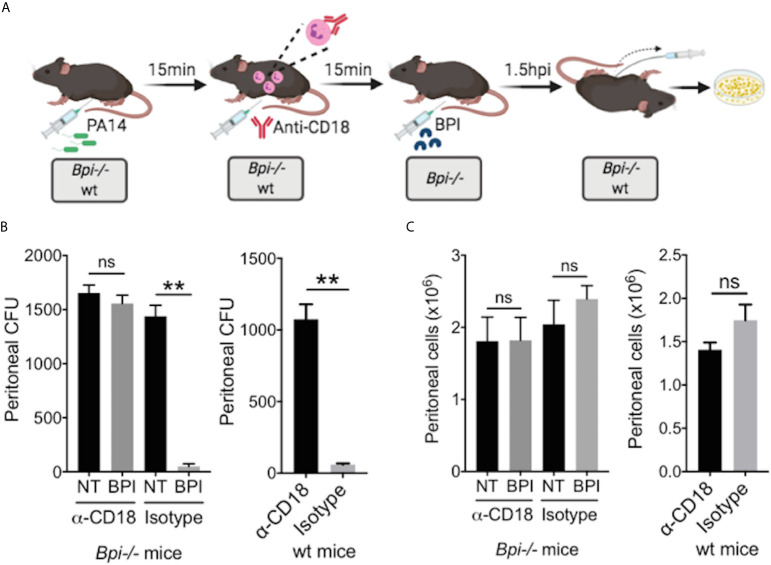
BPI mediates *P. aeruginosa* clearance *in vivo* preeminently by phagocytosis, dependent on CD18. **(A)** Mice (wt or *Bpi-/-*) were infected with 3x10^6^ CFU *P. aeruginosa* PA14 15mins prior to anti-CD18 antibody administration (50μg), *via* intraperitoneal (i.p.) route. In *Bpi-/-* mice, BPI (10μg) was then administered i.p. 15mins following the antibody treatment. Peritoneal lavage was performed 1.5hrs post-anti-CD18 IgG (wt mice) or post-BPI (*Bpi-/-* mice) injection, and plated on LB agar for colony counts. **(B)** Bacterial colony forming units (CFU) recovered from peritoneal fluid after *P. aeruginosa* infection of *Bpi-/-* mice (left panel) treated with a-CD18 IgG or isotype IgG in the absence (NT) or presence of exogenous BPI (n=3 per treatment group), or wt mice (right panel) treated with a-CD18 IgG or isotype IgG (n=3). **(C)** Peritoneal immune cell count in anti-CD18 IgG or isotype treated mice with *P. aeruginosa* infection, with or without BPI administration in *Bpi*-/- mice (n=3). Data were analyzed by paired t-test with parametric test; **p < 0.01, ns, not significant; Error bars represent mean ± SEM.

While prior studies of BPI function, largely limited to *in vitro* systems, have stressed its direct cytocidal activity (15, 19, 20), our data suggest an important role for BPI-mediated phagocytosis in the clearance of GNB *in vivo* (**Figure 4**). To determine whether the exogenous BPI mediates *P. aeruginosa* clearance *in vivo via* phagocytosis or direct killing, we blocked the ability of neutrophils to phagocytes *P. aeruginosa* prior to treatment with hBPI by neutralizing CD18, a β2 integrin required for the uptake of *P. aeruginosa* by phagocytes (33). Anti-CD18 blocking antibody (clone M18/2) or isotype control was given 15 minutes following *P. aeruginosa* (PA14) infection *via* intraperitoneal route; *Bpi-/-* mice received hBPI 15mins after antibody treatment, and peritoneal lavage was performed to determine PA14 CFUs (**Figure 5A**). Anti-CD18 treatment completely abolished the enhanced PA14 clearance mediated by administration of exogenous hBPI (**Figure 5B**). We validated the ability of anti-CD18 antibody to effectively block phagocytosis by treating PA14 infected wt mice and demonstrating higher CFU counts in the lavage, compared to isotype treated controls (**Figure 5B**). Peritoneal immune cell count in both *Bpi-/-* and wt mice was comparable between all treatments (**Figure 5C**), suggesting that the effect seen on peritoneal CFUs (**Figure 5B**) was not due to disparities in immune cell recruitment. *Pseudomonas* strains with T3SS can manipulate host cells leading to evasion of phagocytosis and clearance (34). Infecting mice with *P. aeruginosa* strain deficient in Type III secretion system (T3SS) translocon (PA14ΔpopB) showed comparable recovered CFU to mice infected with *P. aeruginosa* strain sufficient in T3SS translocon (PA14wt) (**Supplemental Figure 11**). These two strains of *P. aeruginosa* are equally susceptible to BPI killing *in vitro* (**Supplemental Figure 12**). Thus, absence or presence of a functional T3SS exhibited no significant effect on *Pseudomonas* CFU in wt and *Bpi-/-* mice. Together, these data suggest that BPI mediates *P. aeruginosa* clearance *in vivo* mainly *via* bacterial phagocytosis and that this process is dependent on CD18.

## Discussion

The selective ability of *P. aeruginosa* to chronically infect the airways in patients with CF, BE and COPD in the absence of neutropenia is poorly understood (5–10). We report that BPI-deficient mice exhibit decreased ability to limit *P. aeruginosa* infection in both the lung and peritoneum *in vivo.* Thus, in this model system, BPI plays a functionally non-redundant role in innate immunity against *P. aeruginosa.* This defect is characterized by increased neutrophil accumulation and production of inflammatory cytokines at sites of infection, propagated by increased recruitment of neutrophils from the bone marrow in *Bpi*-/- mice. The absence of BPI did not impair neutrophil degranulation or ROS production. Rather, *in vitro* and *in vivo* studies demonstrated that *Bpi-/-* neutrophils exhibited markedly decreased phagocytosis of *P. aeruginosa.* This defect was corrected *in vivo* by administering exogenous human BPI. Following phagocytosis, ingested *P. aeruginosa* in neutrophils co-localized with exogenous BPI, suggesting a specific and direct role of intact BPI in bacterial opsonization. Lastly, the inability of exogenous BPI to clear *P. aeruginosa* in the presence of CD18 blockade supports the pre-eminent role of opsonization by BPI as well as identifies its transduction *via* a CD18-dependent pathway.

The anti-bacterial functions of BPI have mainly been studied *in vitro* in systems without cell interactions (14–17, 19, 20, 31, 32). We confirmed that BPI exhibits direct cytocidal activity towards *P. aeruginosa in vitro* (**Supplemental Figure 10**). These studies do not recapitulate the complexity and heterogeneity of the *in vivo* immune cell environment. Our study of BPI-deficient mice is novel, as it is the first study to demonstrate the function of BPI in *P. aeruginosa* phagocytosis and clearance *in vivo*. Prior *in vitro* studies have shown that BPI cytotoxic activities reside in the N-terminal domain which also confers LPS binding (19, 20, 31, 32). In contrast, the C-terminal domain is thought to confer opsonic activity of *Escherichia coli* through an as-yet-undefined pathway in neutrophils and monocytes in *in vitro* systems (17, 35). For the first time, we provide *in vivo* evidence of BPI-mediated phagocytosis, by demonstrating *P. aeruginosa* co-localization with intact human BPI (confirmed by immunofluorescence) in *Bpi-/-* neutrophils in peritoneal infection. Immunoblotting of neutrophil lysates confirmed the absence of BPI proteolysis with neutrophil uptake, suggesting the requirement for intact BPI. Thus, our data strongly support the model by which the cationic N-terminal portion of intact BPI binds LPS on *P. aeruginosa* while the C-terminal domain mediates neutrophil phagocytosis of an intact BPI-*P. aeruginosa* complex. Moreover, CD18 blockade inhibits this activity of exogenous human BPI *in vivo*, thereby confirming that the functional importance of BPI in this model is largely opsonic in nature, rather than by transducing bacterial cytotoxicity.

The ability of CD18 neutralizing antibodies to inhibit BPI-dependent phagocytosis in *Bpi*-/- neutrophils suggests a direct interaction. Recent study has shown that CD18 expression facilitates the uptake of both motile and nonmotile *P. aeruginosa* strains (33). This central role in *P. aeruginosa* uptake has been confirmed; CD18 is the only phagocytic cell surface receptor with genetic evidence to support that it is critical for uptake of *P. aeruginosa* (36, 37). CD18 (β2 integrin) forms heterodimers with CD11a, CD11b, CD11c and CD11d (38). Thus, two models are possible: i) BPI alters *P. aeruginosa* membrane structure to allow for CD18 binding, or ii) BPI directly engages with CD18 for binding and phagocytosis of *P. aeruginosa*. Therefore, our findings of the key importance of BPI in mediating *P. aeruginosa* phagocytosis provide a strong rationale to investigate the CD18-BPI interactions in chronic infectious diseases such as CF, non-CF BE, and COPD, where reduced phagocytosis has been reported (39–43). In CF and non-CF BE, decreased phagocytosis by both neutrophils and macrophages has been identified and correlated with reduced expression of phagocytic receptors (39–41). Whether and how this might influence BPI mediated-phagocytosis warrants investigation. In COPD patients, who frequently suffer from airway *P. aeruginosa* infection (10, 44), macrophages demonstrate significantly reduced phagocytic capacity of bacteria and apoptotic cells (42, 43, 45). Since BPI is also expressed by human macrophages albeit at lower levels than neutrophils (14, 46), BPI is likely to play an important role in macrophage-mediated phagocytosis. Together, our data suggest BPI as a potential therapy to enhance immune cell-mediated phagocytosis in diseases such as CF, BE, and COPD.

The observation of a BPI-specific defect in clearing *P. aeruginosa* is notable given its ubiquitous and chronic nature in CF, BE, and COPD. *P. aeruginosa* infection in these diseases is strongly linked with autoantibodies to BPI (5–10, 47). Moreover, autoantibodies to BPI correlate with worsening lung function in patients with these diseases (5–7). While autoantibodies to BPI are found in other disease states, high-avidity autoantibodies are restricted to patients with chronic lung infection with *P. aeruginosa* (6, 48). Given our data that BPI is required for *P. aeruginosa* immunity *in vivo*, the serologic findings in CF, BE, or COPD suggest that autoantibodies against BPI might shape a protective niche that enables chronic infection by *P. aeruginosa* in the clinical setting. The unique properties of this model not only derive from the specific association of lung infection by *P. aeruginosa* with BPI autoimmunity but also our demonstration of the importance of BPI in clearing this organism *in vivo.* Interestingly, no relationship was seen between circulating BPI levels and antibodies to *P. aeruginosa* in a cohort of bacteremic patients (48). This evidence suggests that patients who are susceptible to *P. aeruginosa* infections are not defective in producing or releasing BPI to combat infections. This further supports the notion that the presence of autoantibodies to BPI restricts its function during infection. In addition to bacterial clearance, the therapeutic potential of BPI is also to modulate inflammation, as phagocytosis in its nature is an anti-inflammatory process (49). Excessive inflammation leads to lung injury in CF (50) particularly by neutrophils. Our data strongly implicate BPI to be a potent anti-inflammatory molecule, as seen with the levels of cytokines *in vivo* and lung pathology. This is particularly important in the light of possible diminished BPI function in patients with anti-BPI autoantibodies. Studies which confirm a functional role of BPI autoantibodies as well as address their specific association with *P. aeruginosa* lung infection would potentially lead to novel immunomodulatory strategies.

## Data Availability Statement

The raw data supporting the conclusions of this article will be made available by the authors, without undue reservation.

## Ethics Statement

The animal study protocols were reviewed, approved, and conducted according to Dartmouth College’s Institutional Animal Care and Use Committee (IACUC Protocol 00002007).

## Author Contributions

JT, WR, and SS-G conceived the project and designed the experiments. JT and RW performed the experiments. AB produced and purified essential reagents. JT and SS-G performed data analyses and interpretation. JT, WR, and SS-G wrote the manuscript. JT prepared the figures. WR supervised all aspects of the study. All authors contributed to the article and approved the submitted version.

## Funding

This work was supported by the Cystic Fibrosis Foundation (CFF) [grant number: RIGBY 20G0], and the BioMT Institute for Biomolecular Targeting core lab (grant: P20GM113132). Further support came from the Translational Research Core at the Dartmouth Geisel School of Medicine, the Shared Resource facility, Irradiation, the Pre-clinical Imaging and Microscopy Resource (IPIMSR) at the Norris Cotton Cancer Center (NCI Cancer Center Support Grant 5P30CA023108-37 and NIH shared instrument grant S10OD21616, for the use of microscopy, pathology and flow cytometry instruments.

## Conflict of Interest

The authors declare that the research was conducted in the absence of any commercial or financial relationships that could be construed as a potential conflict of interest.
