# Supplementary material for: Bactericidal/Permeability-Increasing Protein Preeminently Mediates Clearance of Pseudomonas aeruginosa In Vivo via CD18-Dependent Phagocytosis
Source: Front Immunol. 2021 Apr 26;12:659523. doi: 10.3389/fimmu.2021.659523 (PMC8107240; doi:10.3389/fimmu.2021.659523)
Supplement: Supplementary file 1 [file DataSheet_1.pdf]

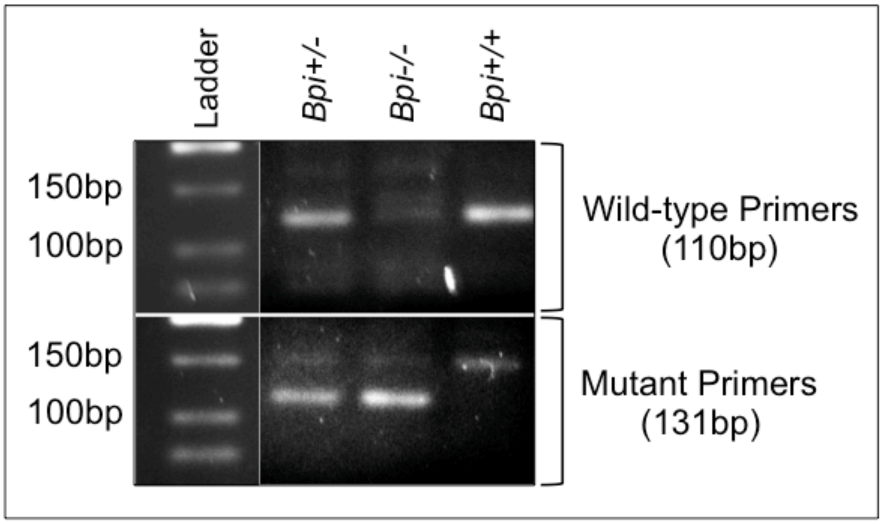

Supplemental Figure 1: Mouse genotype PCR results showing bands (110bp) with wt primers in *Bpi+/-* and *Bpi+/+* mice, and bands (131bp) with BPI-mutant primers in *Bpi+/-* and *Bpi-/-* mice.

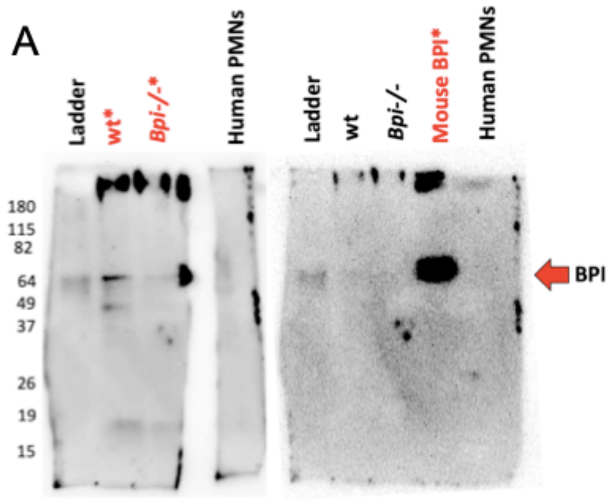

Full unedited blot for Figure 1A

*appeared in the manuscript

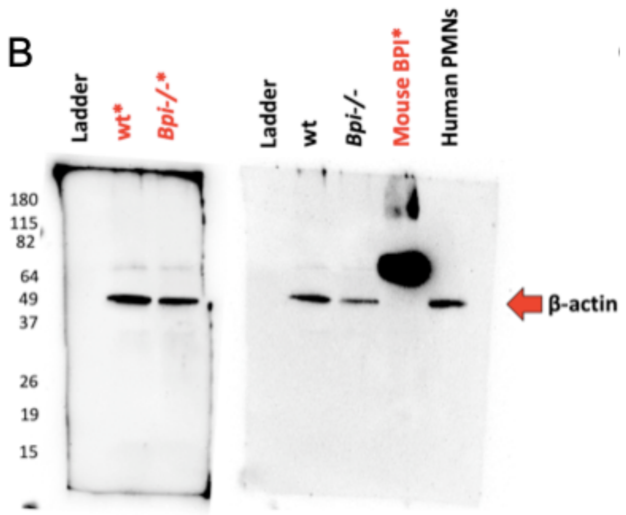

Full unedited blot for Figure 1A

*appeared in the manuscript

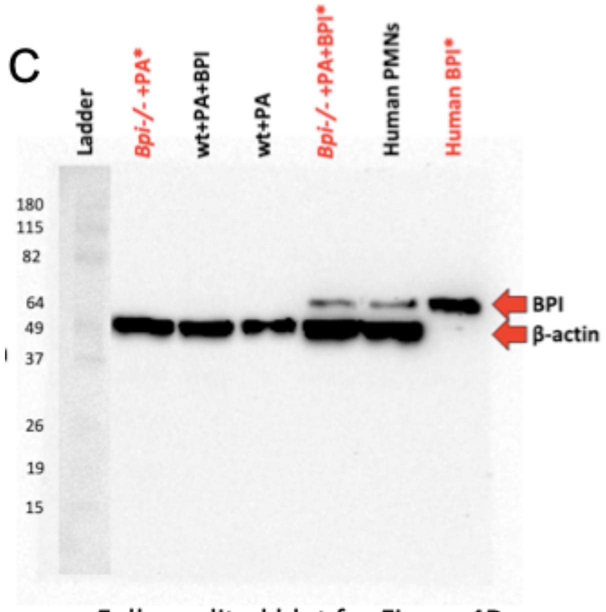

Full unedited blot for Figure 4D

*appeared in the manuscript

Supplemental Figure 2: Full western blot images. (A) Immunoblots of anti-mouse BPI (mBPI) (amino acids 256-269) reactivity to neutrophil lysates (20 µg) from wild type (wt) and BPI-deficient (*Bpi*-/-) mice. Recombinant mBPI (0.5 µg) used as control. **(B)** Immunoblots of anti-beta actin antibody reactivity to neutrophil lysates (20 µg) from wild type (wt) and BPI-deficient (*Bpi*-/-) mice. Recombinant mBPI (0.5 µg) used as control. **(C)** Immunoblot of peritoneal cell lysates (10µg protein, *Bpi-/-*) shows uptake of hBPI with anti-hBPI IgG (amino acids 227-254) following PA14 infection with or without BPI treatment *in vivo*. Anti-beta actin antibody and recombinant mBPI (0.05µg) were used as controls.

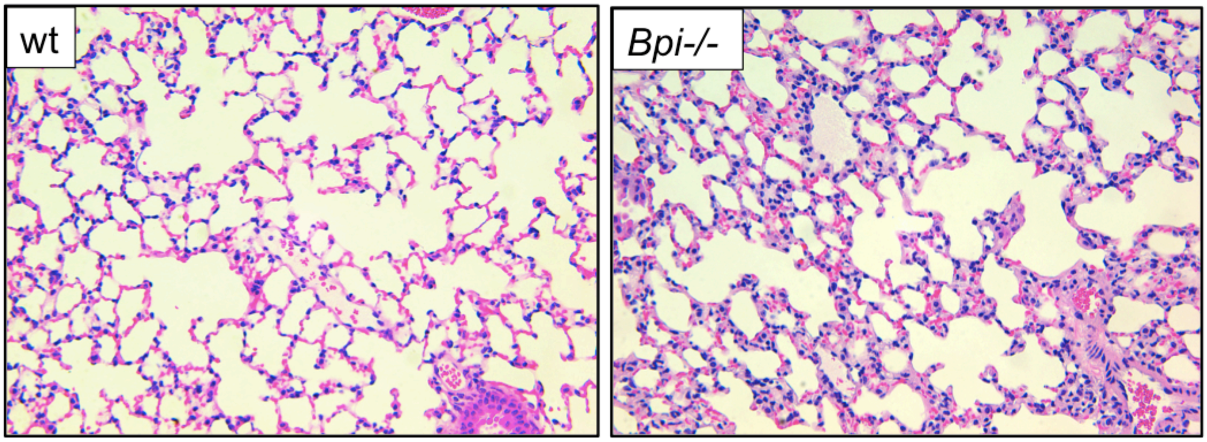

Supplemental Figure 3: Baseline lung pathology of uninfected wt and *Bpi-/-* mice. Representative H&E staining shown, 40X magnification.

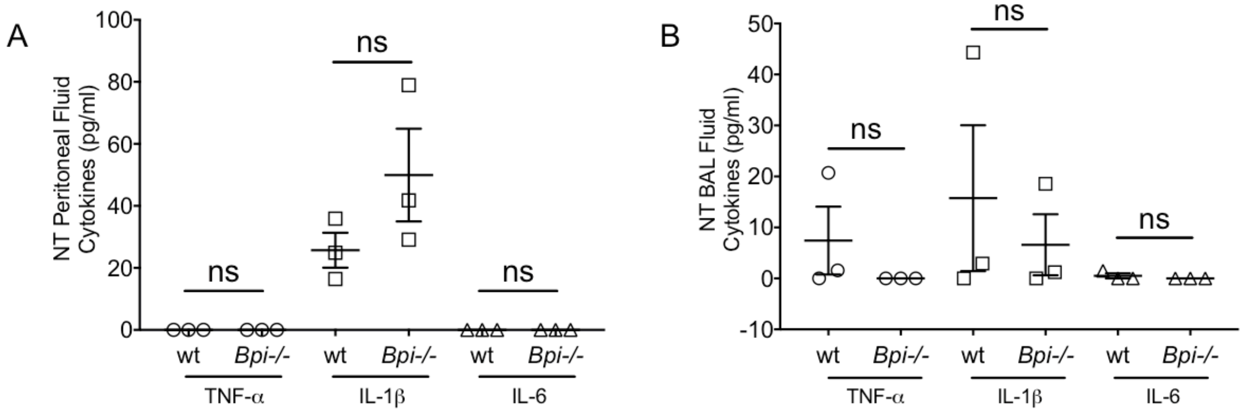

Supplemental Figure 4: Baseline peritoneal and BAL fluid inflammatory cytokine levels in wild type (wt) and BPI-deficient (*Bpi*-/-) mice. (A) Peritoneal fluid cytokines (TNFa, IL-1b, IL-6, pg/ml) measured from mice (n=5) treated with PBS (3hrs) peritoneally. **(B)** Bronchoalveolar lavage (BAL) fluid cytokines (pg/ml) measured from mice (n=5) treated with PBS (3hrs) via oropharyngeal route. NT: no treatment. Data were analyzed by paired t-test; $*p < 0.05$; Error bars represent mean ± SEM.

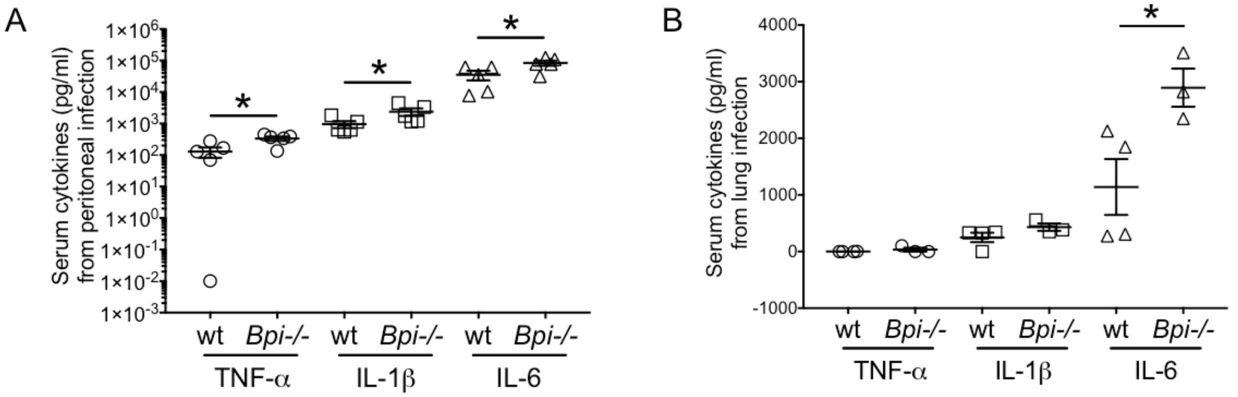

Supplemental Figure 5: Serum inflammatory cytokine levels in wild type (wt) and BPI-deficient (*Bpi*-/-) mice infected with *P. aeruginosa*. (A) Serum cytokines (TNFa, IL-1b, IL-6, pg/ml) measured in mice (n=5) infected with *P. aeruginosa* PA14 ($3x10^6$ CFU, 3hpi) via intraperitoneal route. **(B)** Serum cytokines (pg/ml) measured in mice (n=5) infected with *P. aeruginosa* PA14 ($3x10^6$ CFU, 3hpi) via oropharyngeal route. Data were analyzed by paired t-test; *$p < 0.05$; Error bars represent mean ± SEM.

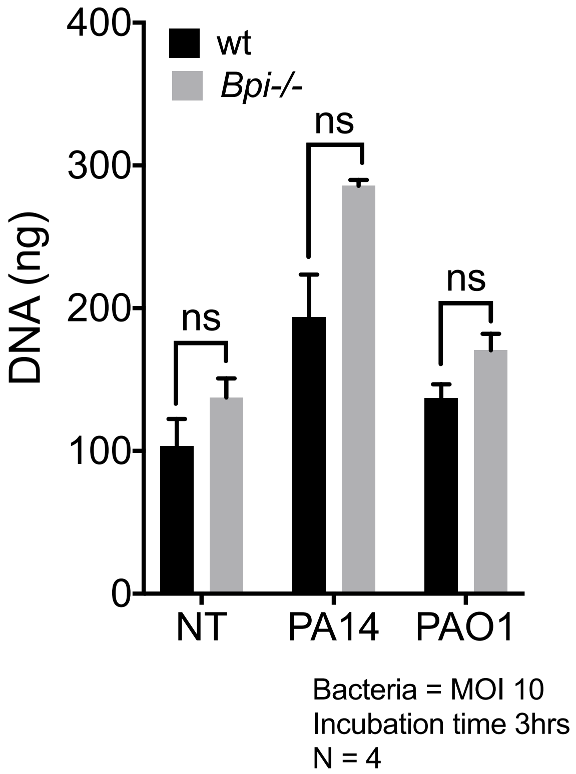

Supplemental Figure 6: No difference in NETosis was observed between wt and *Bpi-/-* neutrophils. Neutrophil extracellular DNA release was shown between wt and *Bpi-/-* neutrophils stimulated with PA14 or PAO1 *P. aeruginosa* MOI10. Data were analyzed by paired t-test; ns = not significant; Error bars represent mean ± SEM.

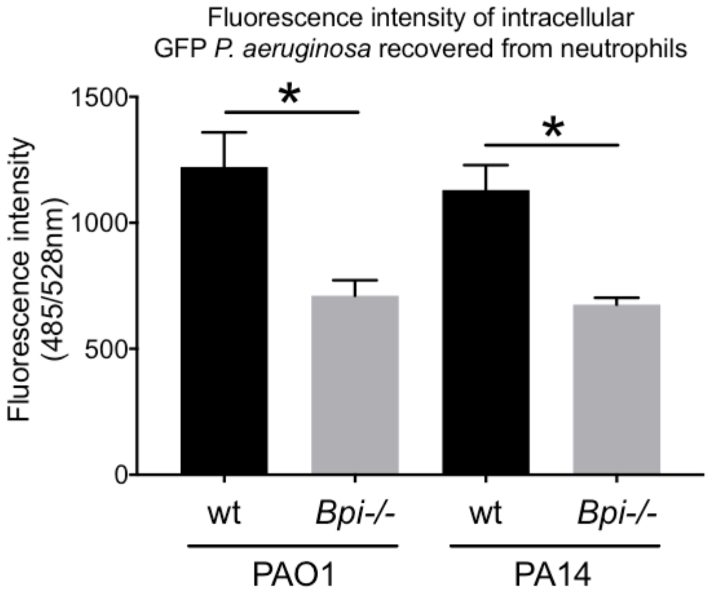

Supplemental Figure 7: BPI is required for efficient phagocytosis of GFP-expressing *P. aeruginosa* PAO1 and PA14 (MOI 10) by neutrophils *in vitro*, as measured by fluorescence intensity (n=3). Data were analyzed by Student t-test; *$p < 0.05$; Error bars represent mean ± SEM.

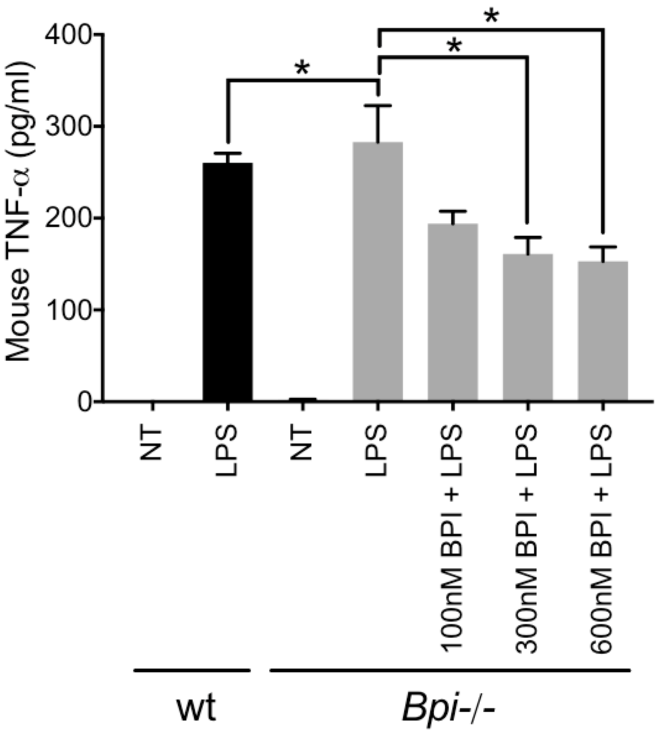

Supplemental Figure 8: Addition of human BPI (hBPI) neutralizes LPS-induced TNFa release in *Bpi-/-* mouse PMNs. Thioglycollate-recruited wild type (wt) or BPI-deficient (*Bpi-/-*) neutrophils were pre-treated with human BPI (hBPI, 0-600nM) before exposed to *E. coli* LPS (0.1ng/ml). Supernatants from *Bpi-/-* mPMNs showed a reduction in TNFa with increased hBPI, in a dose-dependent manner (n=4); data were analyzed by unpaired t-test with Welch's correction. *$p < 0.05$; Error bars represent mean ± SEM.

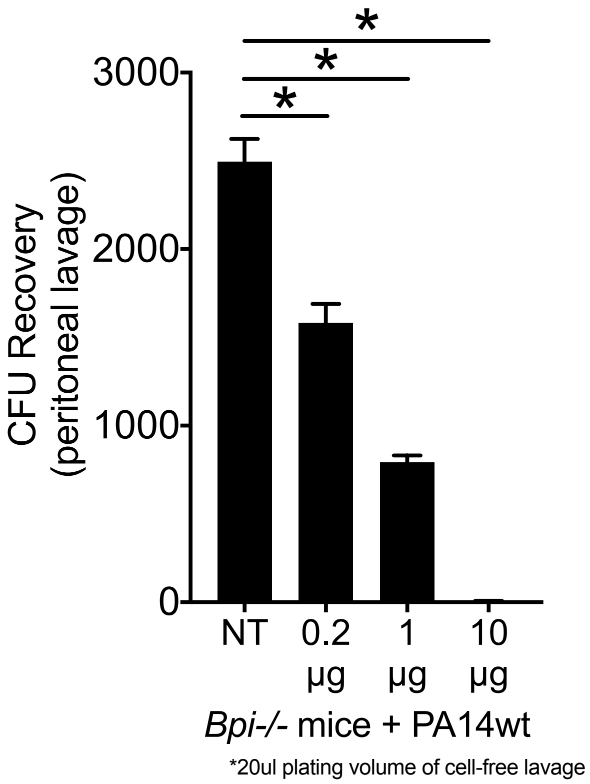

Supplemental Figure 9: Dose-dependent response of BPI in clearing *P. aeruginosa in vivo.* Increasing concentrations of BPI i.p. treatment in *Bpi-/-* mice infected with PA14wt results in reduction in PA14 CFU recovery from cell-free peritoneal lavage. Data were analyzed by paired t-test; *$p < 0.05$; Error bars represent mean ± SEM.

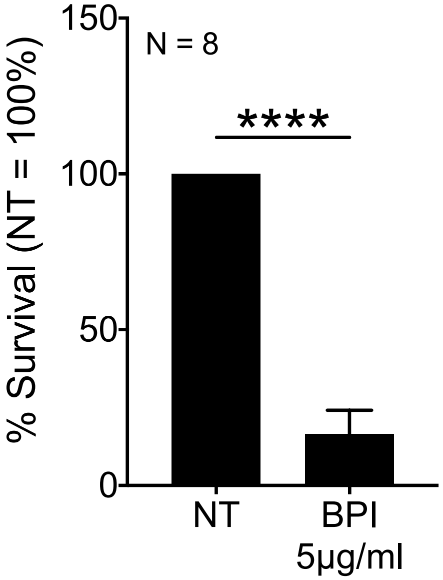

Supplemental Figure 10: BPI exhibits direct cytocidal activity towards *P. aeruginosa in vitro.* The graph represents percent survival of PAO1wt when exposed to BPI at 5ug/ml for 30mins, compared to no BPI treatment counterparts. Data were analyzed by paired t-test; ****$p < 0.0001$; Error bars represent mean ± SEM.

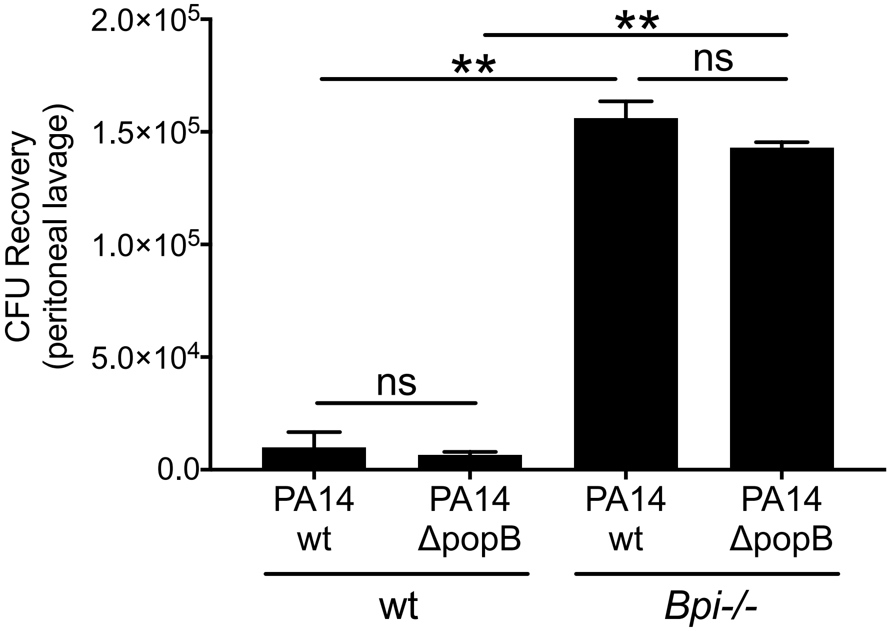

Supplemental Figure 11: Peritoneal lavage CFU recovery from mice (n=2) sufficient (wt) or deficient (*Bpi-/-*) in BPI infected with *P. aeruginosa* strains sufficient (PA14wt) and deficient (PA14ΔpopB) in Type III secretion system (T3SS) translocon. Data were analyzed by paired t-test; **$p < 0.01$; ns = not significant; Error bars represent mean ± SEM.

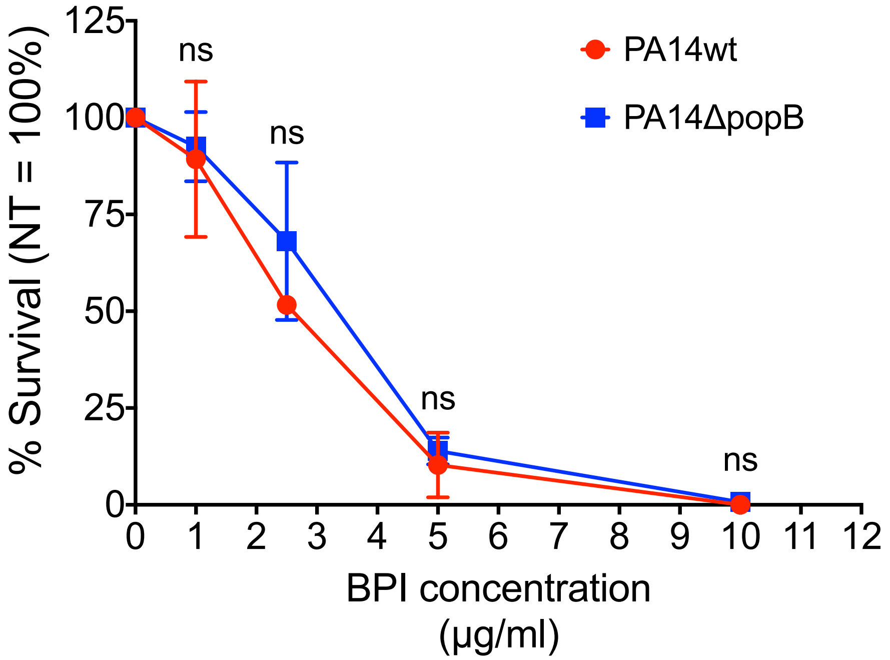

Supplemental Figure 12: Percent survival of *P. aeruginosa* strains sufficient (PA14wt) and deficient (PA14ΔpopB) in Type III secretion system (T3SS) translocon with human BPI treatment (0, 1, 2.5, 5, 10 µg/ml) *in vitro* (n=2). Data were analyzed by paired t-test; ns = not significant; Error bars represent mean ± SEM.
